# Supplementary material for: Effects of different parameters of Tai Chi on the intervention of chronic low back pain: A meta-analysis
Source: PLoS One. 2024 Jul 9;19(7):e0306518. doi: 10.1371/journal.pone.0306518 (PMC11232984; doi:10.1371/journal.pone.0306518)
Supplement: S1 File — (DOCX) [file pone.0306518.s002.docx]

**Search Strategy**

**PubMed**

#1 Search: "Low Back Pain"[Mesh] Sort by: Most Recent

26,116 22:02:05 27285

#2 Search: ((((((((((((((((((((((((((((Back Pain, Low[Title/Abstract]) OR (Back Pains, Low[Title/Abstract])) OR (Low Back Pains[Title/Abstract])) OR (Pain, Low Back[Title/Abstract])) OR (Pains, Low Back[Title/Abstract])) OR (Lumbago[Title/Abstract])) OR (Lower Back Pain[Title/Abstract])) OR (Back Pain, Lower[Title/Abstract])) OR (Back Pains, Lower[Title/Abstract])) OR (Lower Back Pains[Title/Abstract])) OR (Pain, Lower Back[Title/Abstract])) OR (Pains, Lower Back[Title/Abstract])) OR (Low Back Ache[Title/Abstract])) OR (Ache, Low Back[Title/Abstract])) OR (Aches, Low Back[Title/Abstract])) OR (Back Ache, Low[Title/Abstract])) OR (Back Aches, Low[Title/Abstract])) OR (Low Back Aches[Title/Abstract])) OR (Low Backache[Title/Abstract])) OR (Backache, Low[Title/Abstract])) OR (Backaches, Low[Title/Abstract])) OR (Low Backaches[Title/Abstract])) OR (Low Back Pain, Postural[Title/Abstract])) OR (Postural Low Back Pain[Title/Abstract])) OR (Low Back Pain, Posterior Compartment[Title/Abstract])) OR (Low Back Pain, Recurrent[Title/Abstract])) OR (Recurrent Low Back Pain[Title/Abstract])) OR (Low Back Pain, Mechanical[Title/Abstract])) OR (Mechanical Low Back Pain[Title/Abstract])

44,445 22:06:35 3287

#3 Search: ("Low Back Pain"[Mesh]) OR (((((((((((((((((((((((((((((Back Pain, Low[Title/Abstract]) OR (Back Pains, Low[Title/Abstract])) OR (Low Back Pains[Title/Abstract])) OR (Pain, Low Back[Title/Abstract])) OR (Pains, Low Back[Title/Abstract])) OR (Lumbago[Title/Abstract])) OR (Lower Back Pain[Title/Abstract])) OR (Back Pain, Lower[Title/Abstract])) OR (Back Pains, Lower[Title/Abstract])) OR (Lower Back Pains[Title/Abstract])) OR (Pain, Lower Back[Title/Abstract])) OR (Pains, Lower Back[Title/Abstract])) OR (Low Back Ache[Title/Abstract])) OR (Ache, Low Back[Title/Abstract])) OR (Aches, Low Back[Title/Abstract])) OR (Back Ache, Low[Title/Abstract])) OR (Back Aches, Low[Title/Abstract])) OR (Low Back Aches[Title/Abstract])) OR (Low Backache[Title/Abstract])) OR (Backache, Low[Title/Abstract])) OR (Backaches, Low[Title/Abstract])) OR (Low Backaches[Title/Abstract])) OR (Low Back Pain, Postural[Title/Abstract])) OR (Postural Low Back Pain[Title/Abstract])) OR (Low Back Pain, Posterior Compartment[Title/Abstract])) OR (Low Back Pain, Recurrent[Title/Abstract])) OR (Recurrent Low Back Pain[Title/Abstract])) OR (Low Back Pain, Mechanical[Title/Abstract])) OR (Mechanical Low Back Pain[Title/Abstract]))

49,946 22:06:57 47197

#4 Search: "Tai Ji"[Mesh] Sort by: Most Recent

1,429 22:08:40 267

#5 Search: (((((((((Tai-ji[Title/Abstract]) OR (Tai Chi[Title/Abstract])) OR (Chi, Tai[Title/Abstract])) OR (Tai Ji Quan[Title/Abstract])) OR (Ji Quan, Tai[Title/Abstract])) OR (Quan, Tai Ji[Title/Abstract])) OR (Taiji[Title/Abstract])) OR (Taijiquan[Title/Abstract])) OR (T'ai Chi[Title/Abstract])) OR (Tai Chi Chuan[Title/Abstract])

2,487 22:10:24 768

#6 Search: ("Tai Ji"[Mesh]) OR ((((((((((Tai-ji[Title/Abstract]) OR (Tai Chi[Title/Abstract])) OR (Chi, Tai[Title/Abstract])) OR (Tai Ji Quan[Title/Abstract])) OR (Ji Quan, Tai[Title/Abstract])) OR (Quan, Tai Ji[Title/Abstract])) OR (Taiji[Title/Abstract])) OR (Taijiquan[Title/Abstract])) OR (T'ai Chi[Title/Abstract])) OR (Tai Chi Chuan[Title/Abstract]))

2,584 22:10:31 2697

#7 Search: randomized controlled trial[Publication Type] OR randomized[Title/Abstract] OR placebo[Title/Abstract]

995,558 22:10:47 818468

#8 Search: ((("Low Back Pain"[Mesh]) OR (((((((((((((((((((((((((((((Back Pain, Low[Title/Abstract]) OR (Back Pains, Low[Title/Abstract])) OR (Low Back Pains[Title/Abstract])) OR (Pain, Low Back[Title/Abstract])) OR (Pains, Low Back[Title/Abstract])) OR (Lumbago[Title/Abstract])) OR (Lower Back Pain[Title/Abstract])) OR (Back Pain, Lower[Title/Abstract])) OR (Back Pains, Lower[Title/Abstract])) OR (Lower Back Pains[Title/Abstract])) OR (Pain, Lower Back[Title/Abstract])) OR (Pains, Lower Back[Title/Abstract])) OR (Low Back Ache[Title/Abstract])) OR (Ache, Low Back[Title/Abstract])) OR (Aches, Low Back[Title/Abstract])) OR (Back Ache, Low[Title/Abstract])) OR (Back Aches, Low[Title/Abstract])) OR (Low Back Aches[Title/Abstract])) OR (Low Backache[Title/Abstract])) OR (Backache, Low[Title/Abstract])) OR (Backaches, Low[Title/Abstract])) OR (Low Backaches[Title/Abstract])) OR (Low Back Pain, Postural[Title/Abstract])) OR (Postural Low Back Pain[Title/Abstract])) OR (Low Back Pain, Posterior Compartment[Title/Abstract])) OR (Low Back Pain, Recurrent[Title/Abstract])) OR (Recurrent Low Back Pain[Title/Abstract])) OR (Low Back Pain, Mechanical[Title/Abstract])) OR (Mechanical Low Back Pain[Title/Abstract]))) AND (("Tai Ji"[Mesh]) OR ((((((((((Tai-ji[Title/Abstract]) OR (Tai Chi[Title/Abstract])) OR (Chi, Tai[Title/Abstract])) OR (Tai Ji Quan[Title/Abstract])) OR (Ji Quan, Tai[Title/Abstract])) OR (Quan, Tai Ji[Title/Abstract])) OR (Taiji[Title/Abstract])) OR (Taijiquan[Title/Abstract])) OR (T'ai Chi[Title/Abstract])) OR (Tai Chi Chuan[Title/Abstract])))) AND (randomized controlled trial[Publication Type] OR randomized[Title/Abstract] OR placebo[Title/Abstract])

35 22:11:39 39

**Embase**

#1

'back pain, low':ab,ti OR 'back pains, low':ab,ti OR 'low back pains':ab,ti OR 'pain, low back':ab,ti OR 'pains, low back':ab,ti OR 'lumbago':ab,ti OR 'lower back pain':ab,ti OR 'back pain, lower':ab,ti OR 'back pains, lower':ab,ti OR 'lower back pains':ab,ti OR 'pain, lower back':ab,ti OR 'pains, lower back':ab,ti OR 'low back ache':ab,ti OR 'ache, low back':ab,ti OR 'aches, low back':ab,ti OR 'back ache, low':ab,ti OR 'back aches, low':ab,ti OR 'low back aches':ab,ti OR 'low backache':ab,ti OR 'backache, low':ab,ti OR 'backaches, low':ab,ti OR 'low backaches':ab,ti OR 'low back pain, postural':ab,ti OR 'postural low back pain':ab,ti OR 'low back pain, posterior compartment':ab,ti OR 'low back pain, recurrent':ab,ti OR 'recurrent low back pain':ab,ti OR 'low back pain, mechanical':ab,ti OR 'mechanical low back pain':ab,ti 8,861

#2

low AND back AND pain 86,505

#3

#1 OR #2 88,566

#4

'tai-ji':ab,ti OR 'chi, tai':ab,ti OR 'tai ji quan':ab,ti OR 'ji quan, tai':ab,ti OR 'quan, tai ji':ab,ti OR 'taiji':ab,ti OR 'taijiquan':ab,ti OR 'tai chi':ab,ti OR 'tai chi chuan':ab,ti 3,292

#5

tai AND ji 2,705

#6

#4 OR #5 5,852

#7

'randomized controlled trial':ab,ti OR 'randomized':ab,ti OR 'placebo':ab,ti 1,102,152

#8

#3 AND #6 AND #7 31

**Cochrane**

#1 (Low Back Pain):ab,ti,kw OR (Back Pain, Low):ab,ti,kw OR (Back Pains, Low):ab,ti,kw OR (Low Back Pains):ab,ti,kw OR (Pain, Low Back):ab,ti,kw OR (Pains, Low Back):ab,ti,kw OR (Lumbago):ab,ti,kw OR (Lower Back Pain):ab,ti,kw OR (Back Pain, Lower):ab,ti,kw OR (Back Pains, Lower):ab,ti,kw OR (Lower Back Pains):ab,ti,kw OR (Pain, Lower Back):ab,ti,kw OR (Pains, Lower Back):ab,ti,kw OR (Low Back Ache):ab,ti,kw OR (Ache, Low Back):ab,ti,kw OR (Aches, Low Back):ab,ti,kw OR (Back Ache, Low):ab,ti,kw OR (Back Aches, Low):ab,ti,kw OR (Low Back Aches):ab,ti,kw OR (Low Backache):ab,ti,kw OR (Backache, Low):ab,ti,kw OR (Backaches, Low):ab,ti,kw OR (Low Backaches):ab,ti,kw OR (Low Back Pain, Postural):ab,ti,kw OR (Postural Low Back Pain):ab,ti,kw OR (Low Back Pain, Posterior Compartment):ab,ti,kw OR (Low Back Pain, Recurrent):ab,ti,kw OR (Recurrent Low Back Pain):ab,ti,kw OR (Low Back Pain, Mechanical):ab,ti,kw OR (Mechanical Low Back Pain):ab,ti,kw 15905

#2 (Tai Ji):ab,ti,kw OR (Tai-ji):ab,ti,kw OR (Tai Chi):ab,ti,kw OR (Chi, Tai):ab,ti,kw OR (Tai Ji Quan):ab,ti,kw OR (Ji Quan, Tai):ab,ti,kw OR (Quan, Tai Ji):ab,ti,kw OR (Taiji):ab,ti,kw OR (Taijiquan):ab,ti,kw OR (T'ai Chi):ab,ti,kw OR (Tai Chi Chuan):ab,ti,kw 1760

#3 (randomized controlled trial):ab,ti,kw OR (Randomized):ab,ti,kw OR (placebo):ab,ti,kw 1175867

#4 #1 AND #2 AND #3 29 35

**WOS**

1

TS=(Low Back Pain OR Back Pain, Low OR Back Pains, Low OR Low Back Pains OR Pain, Low Back OR Pains, Low Back OR Lumbago OR Lower Back Pain OR Back Pain, Lower OR Back Pains, Lower OR Lower Back Pains OR Pain, Lower Back OR Pains, Lower Back OR Low Back Ache OR Ache, Low Back OR Aches, Low Back OR Back Ache, Low OR Back Aches, Low OR Low Back Aches OR Low Backache OR Backache, Low OR Backaches, Low OR Low Backaches OR Low Back Pain, Postural OR Postural Low Back Pain OR Low Back Pain, Posterior Compartment OR Low Back Pain, Recurrent OR Recurrent Low Back Pain OR Low Back Pain, Mechanical OR Mechanical Low Back Pain) 49,443

2

TS=(Tai Ji OR Tai-ji OR Tai Chi OR Chi, Tai OR Tai Ji Quan OR Ji Quan, Tai OR Quan, Tai Ji OR Taiji OR Taijiquan OR T'ai Chi OR Tai Chi Chuan) 3,832

3

TS=(randomized controlled trial OR Randomized OR placebo) 891,035

4

#1 AND #2 AND #3 82 89

**知网**

1 腰痛、下腰痛、下腰疼、下背痛、腰背痛、腰肌劳损、腰腿痛 2.94万 3.04万

AND

2 太极拳、太极 84 91

AND

3 随机对照试验、 随机对照实验、随机对照研究、随机对照、RCT、随机 8 9

**万方**

1 腰痛 OR 下腰痛 OR 下腰疼 OR 下背痛 OR 腰背痛 OR 腰肌劳损 OR 腰腿痛

AND

2 太极拳 OR 太极

AND

3 随机对照试验 OR 随机对照实验 OR 随机对照研究 OR 随机对照 OR RCT OR 随机 29 33

**维普**

1 腰痛、下腰痛、下腰疼、下背痛、腰背痛、腰肌劳损、腰腿痛

AND

2 太极拳、太极

AND

3 随机对照试验、 随机对照实验、随机对照研究、随机对照、RCT、随机 14 17

**CBM**

1 "腰痛"[不加权:扩展] 8349 10:16:27 8347

2 "下腰痛"[常用字段:智能] OR "下背痛"[常用字段:智能] OR "腰背痛"[常用字段:智能] OR "腰肌劳损"[常用字段:智能] OR "腰腿痛"[常用字段:智能] OR "下腰疼"[常用字段:智能] 20094 10:17:49 20724

3 (#2) OR (#1) 25408 10:17:59 26037

4 "太极拳"[不加权:扩展] 1287 10:18:17 1286

5 "太极"[常用字段:智能] 2565 10:18:44 2668

6 (#5) OR (#4) 2565 10:18:53 2668

7 "随机对照试验"[不加权:扩展] 197012 10:19:40 196499

8 "随机对照实验"[常用字段:智能] OR "随机对照研究"[常用字段:智能] OR "随机对照"[常用字段:智能] OR "RCT"[常用字段:智能] OR "随机"[常用字段:智能] 1846633 10:20:36 1918454

9 (#8) OR (#7) 1846832 10:20:50 1918669

10 (#9) AND (#6) AND (#3) 18 10:21:11 19
