# Supplementary material for: Effects of different parameters of Tai Chi on the intervention of chronic low back pain: A meta-analysis
Source: PLoS One. 2024 Jul 9;19(7):e0306518. doi: 10.1371/journal.pone.0306518 (PMC11232984; doi:10.1371/journal.pone.0306518)
Supplement: Attachment — Submitted filename: Response to reviewer.doc [file pone.0306518.s004.doc]

Date: Oct.18, 2023

**Covering letter**

**Dear Editor-in-Chief,**

It was mentioned in the abstract that the date retrieval was on May 2022, but in fact May 2023 mentioned in the part of 2.2, that is to say that the abstract was a clerical error. I'm sorry for the trouble caused to your work.

And in this manuscript, we searched the literature from May 2023 to the present October 2023. After 8 duplicates were removed, 17 articles were screened for eligibility. By reviewing the titles, abstracts, and full content of the selected studies, we excluded an additional 16 articles (review = 4, protocol = 5, non-chronic low back pain = 3, non-TCE =2, no primary outcome =2). Finally, this review included one additional randomized controlled trial.

Best wishes!

Corresponding Author: Min Yang

Tel: +8615603370295

Postal code: 066000
