# Supplementary material for: Effects of different parameters of Tai Chi on the intervention of chronic low back pain: A meta-analysis
Source: PLoS One. 2024 Jul 9;19(7):e0306518. doi: 10.1371/journal.pone.0306518 (PMC11232984; doi:10.1371/journal.pone.0306518)
Supplement: Attachment — Submitted filename: Review of manuscript D-23-34184.docx [file pone.0306518.s005.docx]

Review of manuscript D-23-34184:

Effects of different parameters of Tai Chi on the intervention of chronic low back pain: a meta-analysis

Thank you for the opportunity to read the presented topics. The authors addressed the important issue of analyzing the impact of various variants of Tai Chi exercises on the level of back pain.

The meta-analysis method was used for evaluation, including manuscripts published in Chinese and English.

In terms of the methodology used, the work was prepared correctly and the procedure used was also correct.

In terms of formal preparation of the work, I find some shortcomings:

1. The literature presented in table 1 (line 209) needs to be sorted, the layout should be arranged according to an ordinal variable (year of publication) and the width of the columns should be adjusted so that the data in the columns is legible. The numbering of items [37], despite explanations in the work, should be adapted to their appearance in the text.

2. The provisions regarding the presentation of analysis results in the text should be unified; please adopt uniform provisions, mainly the use or intervals between the results.

Example:

Line 147-148

Line 235-236 [WMD (95% CI) = - 0.85 (-1.10, -0.60), p < 0.000],

Proposed entry [WMD (95% CI) = -0.85 (-1.10, -0.60), p<0.000],

Line 244-246 suggested entry [WMD (95% CI) = -0.69 (-1.89, -0.50), p=0.26],

Line 272 -273

Please correct the entire manuscript in this respect, the work is an interesting aspect of the impact of Tai Chi exercises and these shortcomings largely make it illegible and less accessible to the reader.

A good example of a study is a work prepared using the same method:

Liang T, Zhang X, Wang Y et al. Intervention effect of taijiquan exercises on the ankle joint of the elderly – meta analysis. ARCH BUDO. 2023;19

Archives of Budo - Abstract (archbudo.com)

I hope that the comments I have presented will be helpful to you.
