# Supplementary material for: Effects of different parameters of Tai Chi on the intervention of chronic low back pain: A meta-analysis
Source: PLoS One. 2024 Jul 9;19(7):e0306518. doi: 10.1371/journal.pone.0306518 (PMC11232984; doi:10.1371/journal.pone.0306518)
Supplement: Attachment — Submitted filename: Response to Reviewers.docx [file pone.0306518.s006.docx]

Dear Editors and Reviewers,

Hello!

We sincerely thank you for all the effort you have put into our manuscript and for your constructive comments and suggestions, which have greatly improved the quality of our manuscript. We have considered the suggestions and comments carefully and made some revisions. Revision note, point by point, are given as follows:

**Reviewer #1:**

ABSTRACT
1. The risk of bias and GRADE findings were not reported.

Response: Thank you very much for your careful review. We have added the risk of bias and GRADE findings in the abstract in line 38,48-50, Page 2 of the revised manuscript.

METHODS

1.Literature retrieval strategy: You need to present the complete search strategy for at least one database so that people can replicate your search terms if they intend to do so. You just presented a very rough search strategy without indicating which database it was.

Response: Thank you very much for your valuable suggestion, and we have added the PubMed search strategy in Box 1, Page 5 of the revised manuscript.

2.Inclusion criteria: Did you include all comparators for this review? The comparison groups were not mentioned at all.

Response: Thanks for your careful consideration. We sorted out the literature characteristic in Table 1 and deleted part of the comparison groups that were not relevant to this study.

3.Secondary outcomes: What did you mean by the assessment of dysfunction? Did you mean disability/functional limitations?

Response: The dysfunction did mean disability/functional limitations in the review, and the relevant expression has been revised.

4.How did you extract studies? How did you resolve disagreements?

Response: We are deeply sorry for the inconvenience caused to the reviewers. In this study, two evaluators independently searched the studies. Firstly, those studies meeting the research purpose were selected by reading the title and abstract. Then, after the full texts were carefully read, the final screening and review were conducted according to the inclusion and exclusion criteria. Meanwhile, we also cross-checked, and extracted the data uniformly, with the disagreements resolved by discussion and group review. We have added the relevant content to the section 2.4 Literature Screening and data extraction in line 134-141, Page 6 of the revised manuscript.

5.Statistical methods: How were study groupings performed? Did you check clinical homogeneity? This is very necessary in meta-analysis.

Response: Thank you for your rigorous advice. Considering that the number of included studies meeting the criteria was not large, and the subgroup analysis should not be over-crossed, we finally performed subgroup analysis from the perspective of innovation, such as intervention measures (Tai Chi alone, Tai Chi as an additional therapy, Ai Chi), Tai Chi schools (Chen's, Yang's) and total intervention times (> 30 times and ≤30 times) to explore their impact. Clinical homogeneity was checked by the I² test. See 2.5 Statistical Methods for details in line 157-161, Page 6 of the revised manuscript.

6.Statistical methods: Could you state the criterion that informed your choice for using “fixed effect models” in the meta-analyses? You have stated the criteria for the random effect models.

Response: Thank you very much for your careful examination. When I² < 50% and P > 0.1, it suggested the heterogeneity was low and the fixed effect model was used. See 2.5 Statistical Methods for additional information in line 152-154, Page 6 of the revised manuscript.

RESULTS

1.You excluded 19 irrelevant studies after the full-text screening. Your readers would want to know whether the 1p papers warrant exclusion. You need to present a table of excluded studies with reasons otherwise we will not know if you have performed a thorough review.

Response: We are grateful for your valuable suggestion. And this part is accessible in the attached Excel file.

2. Please indicate in Table 1 where the extracted studies are funded or not funded.

Response: Thank you for your precious suggestion and the funded information was added in the last column of Table 1.

3. In Figure 1, what did you mean by studies included in the review (n=1) and reports of included studies (n=13)? This is not the way of reporting the PRISMA flow chart.

Response: Thanks for your careful examination. The PRISMA flow chart has been revised, and the final number of included studies has also been revised due to updated inclusion and exclusion criteria.

4. I note that you have “experimental and control groups” in your forest plots, and I understand that Tai Chi is the experimental group, however, you failed to clearly state control groups. Were you lumping all other treatments as control groups? If yes, this is not the way to do meta-analyses because you have differing comparators in Table 1 and they all can’t be lumped as “control”. If you do that you are then hiding the effects of other interventions (massage, medication, exercise etc). You need to redo all your meta-analyses.

Response: We fully understand the reviewer’s concerns, and we updated the intervention criteria: 1. Comparison of Tai Chi, Ai Chi and control group (routine care, blank control, placebo); 2. Comparison between Tai Chi combined with other intervention therapy and the other intervention therapy alone (and the other intervention therapy is the same one). Studies other than the above two comparisons were excluded. And there were major changes in the follow-up, especially in the subgroup analysis. Shown in the line 120-123, Page 5 of the revised manuscript.

5. Sensitivity analyses were performed but the criteria were not mentioned.

Response: Thanks very much for your careful examination, and the criteria of Sensitivity analyses is available in 2.5 Statistical Methods in the line 156-157, Page 6 of the revised manuscript.

6. The way you performed the subgroup analyses is very confusing.

Response: We apologize sincerely for the inconvenience caused to the reviewers. The confusion of subgroup analyses may be due to that there was only one study in some groups, and we deleted it together with its group. This time, we reformulated the subgroup analyses criteria, in which the phenomenon would be avoided.

7.You reported adverse events, but you failed to mention this in your primary and secondary outcomes.

Response: We are grateful for your valuable suggestion. The adverse events were classified into the secondary outcomes, which can be seen in line 287-291, page 13 of the revised manuscript.

**Reviewer #2:**

1. The literature presented in table 1 (line 209) needs to be sorted, the layout should be arranged according to an ordinal variable (year of publication) and the width of the columns should be adjusted so that the data in the columns is legible. The numbering of items [37], despite explanations in the work, should be adapted to their appearance in the text.

Response: Thank you very much for your precious suggestion. The problems you mentioned have been revised, which can be seen in Table 1.

2. The provisions regarding the presentation of analysis results in the text should be unified; please adopt uniform provisions, mainly the use or intervals between the results.
Example:

Line 147-148
Line 235-236 [WMD (95% CI) = - 0.85 (-1.10, -0.60), p < 0.000],
Proposed entry [WMD (95% CI) = -0.85 (-1.10, -0.60), p<0.000],
Line 244-246 suggested entry [WMD (95% CI) = -0.69 (-1.89, -0.50), p=0.26],
Line 272 -273

Response: Thanks for your careful examination and we are sorry for our carelessness. Your advice is very clear and we have checked the formatting problems in the text.

**Reviewer #3:**

1.Limited discussion of heterogeneity: While the authors acknowledge the presence of heterogeneity in the analysis, the discussion could be further expanded to explore the potential sources of heterogeneity and their impact on the results.

Response: Thank you very much for your valuable suggestion. This section has some extensions. Please see line 378-385, page 17 of the revised manuscript for details.

2.Lack of subgroup analysis for specific Tai Chi styles: The study investigated the overall effect of Tai Chi, but further analysis for different styles (e.g., Yang style, Sun style) could provide more specific guidance for practitioners.

Response: We are very thankful for your precious suggestion. We made a more detailed analysis about the schools of Tai Chi in the section of Discussion, which can be seen in line 359-378, page 16-17 of the revised manuscript.
3.Limited exploration of potential harms: While the study focuses on the benefits of Tai Chi, a brief discussion of potential adverse effects or contraindications would be beneficial for clinicians.

Response: Thank you very much for your valuable suggestion. We made a more detailed analysis about adverse effects in the section of Discussion, discussing the potential contraindications, which can be seen in line 407-412, Page 18 of the revised manuscript.

Minor comments
* In page 3, line 60, write in words by-avoiding the symbols
* In page 3, line 62, low back discomfort? I think the focus of the study is CLBP. Be specific and use the same terminologies consistently.
*Page number 4, line 67-70, this crucial sentence requires reference from high quality studies
*Please write the full form of PRISMA

Response: Thanks for your careful examination. The problems above have been revised shown in line 64,71and figure 1.

Major comments
1.It would be nice if you can give the complete set of keywords specific to each database as additional material

Response: We are thankful for your precious suggestion. The complete set of keywords specific to each database is in the Appendix Word.
2.I would suggest you to improve the writing in the eligibility criteria section. You might avoid the numbers and write in paragraph form

Response: Thank you very much for your careful examination and we have revised the formatting problem.
3.The intervention included different forms of Tai Chi, such as Tai Chi alone, Tai Chi
131 in combination with other usual treatments, or Ai Chi: in this case, how do you determine if the effect is from tai chai or from the co intervention? what if the whole effect was from the co intervention? how do you deal with this?

Response: Thank you for your careful consideration. Among the updated criteria for interventions, one was a comparison of tai chi combined with other treatments and other treatments alone (the other treatment is the same), which can determine the efficacy of Tai Chi as an additional therapy. Additionally, sensitivity analysis, meta-regression analysis and subgroup analysis were used to explore Tai Chi alone, Tai Chi as additional therapy combined with other treatments, and Ai Chi. The results showed that no significant difference was found between Tai Chi alone and Tai Chi as additional therapy combined with other treatments.
