# Supplementary material for: Effects of different parameters of Tai Chi on the intervention of chronic low back pain: A meta-analysis
Source: PLoS One. 2024 Jul 9;19(7):e0306518. doi: 10.1371/journal.pone.0306518 (PMC11232984; doi:10.1371/journal.pone.0306518)
Supplement: Attachment — Submitted filename: Response to Reviewers.docx [file pone.0306518.s008.docx]

Dear Editors and Reviewers,

Hello!

We sincerely thank you for all the effort you have put into our manuscript and for your constructive comments and suggestions, which have greatly improved the quality of our manuscript.

Firstly, we checked all the included papers and confirmed that no retracted papers were cited.

Secondly, we have considered the suggestions and comments carefully and made some revisions. Revision note, point by point, are given as follows:

Reviewer #2: 
1 you use the "Time new Roman" font, but on lines 153, 155, 161 you use the "SimSun" font. Similarly in Table 2.

3 introduced literature references line 212, 214, 216 are using the SimSun font.

The above two questions are about the format, here is a unified reply. Thank you for your careful guidance, and we are sorry again for our carelessness. We have changed the format of the content you annotated and checked the full text to prevent this problem from happening again.
2 Table 1 requires correction - the proposal was introduced in part of the table (yellow), in the previous version the table was difficult to read.

We do not fully understand this issue. Compared with previous version, we think that what you have said may only mean the total number of interventions is too general, so the intervention cycle, single intervention time and intervention frequency are respectively expressed, and how the total number of interventions is obtained is explained in the text.

4. the basic error indicating lack of care is the prepared list of literature. Positions 12 and 30 are repeated, as are positions 28 and 38.

We have deleted the duplicate documents and revised the corner notes in order.
