# Supplementary material for: Effects of different parameters of Tai Chi on the intervention of chronic low back pain: A meta-analysis
Source: PLoS One. 2024 Jul 9;19(7):e0306518. doi: 10.1371/journal.pone.0306518 (PMC11232984; doi:10.1371/journal.pone.0306518)
Supplement: Attachment — Submitted filename: Response to Reviewers.docx [file pone.0306518.s010.docx]

Dear Editors and Reviewers,

Hello!

We sincerely thank you for all the effort you have put into our manuscript and for your constructive comments and suggestions, which have greatly improved the quality of our manuscript.

**Abstract**
Specify the inclusion criteria for the review instead of mentioning search keywords.

Re: Removed keywords and described the inclusion criteria.
Mention the start date of the database search.

Re: Added, from inception.
Please either mention the names of all the databases that have been searched or delete the names of the two databases that you have mentioned.

Re: The names of all the 8 databases were mentioned here.
Specify the methods used to assess the risk of bias in the included studies.

Re: Added, quality assessment of each study used ROB2.
Mention the unit of inversion time "(> 30 and ≤ 30)". It is not clear whether it is the number of sessions or the duration of each session.

Re: It is the number of total sessions, the full text has been checked and refined.
- Line 53: " Tai Chi and Tai Chi alone": Does first tai chi mean Tai Chi as an additional therapy? Introduction

回复；该处的第一个太极拳指的是单独太极拳，之前语法错误还请见谅。

Re: The first taijiquan there referred to taijiquan alone, and I apologize for the carelessness.
- Line 91: "In recent years in recent years". Words are repeated twice.

Re: Sorry, the duplicate was removed.
**Method**
- In line 105: Literature retrieval strategy- PICO should be considered in the search strategy. However, the outcome/ outcomes are not clear in the provided search strategy.

Re: In this study, pain level was the main indicator, and the search terms related to “low back pain” could represent the retrieval of the results. Therefore, “pain” was not searched further in the search strategy.
- Line 127, 128: "Chinese and English only". It can be written: only Chinese and English language literature were included.

Re: Thank you for your suggestion, it has been switched.
**Discussion**
The discussion is too long. Please summarize the text.

Re: In the discussion, the content was adjusted according to the comments of the previous reviewers, including the synthesized results, tai chi mechanism, subgroup analysis and discussion, secondary endpoint indicators, and study strengths and limitations. And according to this suggestion, we read through the discussion section and simplified some statements.
